# Perilipin 5 Ameliorates Hepatic Stellate Cell Activation via SMAD2/3 and SNAIL Signaling Pathways and Suppresses STAT3 Activation

**DOI:** 10.3390/cells10092184

**Published:** 2021-08-24

**Authors:** Rafael Cierpka, Ralf Weiskirchen, Anastasia Asimakopoulos

**Affiliations:** Institute of Molecular Pathobiochemistry, Experimental Gene Therapy and Clinical Chemistry (IFMPEGKC), RWTH University Hospital Aachen, D-52074 Aachen, Germany; Rafael.Cierpka@rwth-aachen.de

**Keywords:** PLIN5, hepatic stellate cells, hepatic fibrogenesis, quiescence, SMAD2/3, SNAIL, α-SMA, collagen, TGF-β1

## Abstract

Comprehending the molecular mechanisms underlying hepatic fibrogenesis is essential to the development of treatment. The hallmark of hepatic fibrosis is the development and deposition of excess fibrous connective tissue forcing tissue remodeling. Hepatic stellate cells (HSC) play a major role in the pathogenesis of liver fibrosis. Their activation via the transforming growth factor-β1 (TGF-β1) as a key mediator is considered the crucial event in the pathophysiology of hepatic fibrogenesis. It has been shown that Perilipin 5 (PLIN5), known as a lipid droplet structural protein that is highly expressed in oxidative tissue, can inhibit such activation through various mechanisms associated with lipid metabolism. This study aimed to investigate the possible influence of PLIN5 on TGF-β1 signaling. Our findings confirm the importance of PLIN5 in maintaining HSC quiescence in vivo and in vitro. PLIN5 overexpression suppresses the TGF-β1-SMAD2/3 and SNAIL signaling pathways as well as the activation of the signal transducers and activators of transcription 3 (STAT3). These findings derived from experiments in hepatic cell lines LX-2 and Col-GFP, in which overexpression of PLIN5 was able to downregulate the signaling pathways SMAD2/3 and SNAIL activated previously by TGF-β1 treatment. Furthermore, TGF-β1-mediatedinduction of extracellular matrix proteins, such as collagen type I (COL1), Fibronectin, and α-smooth muscle actin (α-SMA), was suppressed by PLIN5. Moreover, STAT3, which is interrelated with TGF-β1 was already basally activated in the cell lines and inhibited by PLIN5 overexpression, leading to a further reduction in HSC activity shown by lowered α-SMA expression. This extension of the intervening mechanisms presents PLIN5 as a potent and pleiotropic target in HSC activation.

## 1. Introduction

Hepatic fibrosis, as a result of chronic liver injury, is a global medical burden with economic strains due to its high prevalence and potential complications [1]. Various triggers, such as alcohol abuse, hepatitis viruses, and metabolic disorders lead to an inflammatory and pro-fibrogenic process with an increased deposition of extracellular matrix (ECM) and overexpression of collagen type I (COL1) and Fibronectin [2], thus leading to tissue scarring. Fibrosis is still a reversible intermediate stage, which when untreated can progress to liver cirrhosis and possibly to hepatocellular carcinoma [3].

The crucial process in the pathophysiology of hepatic fibrogenesis is the activation of hepatic stellate cells (HSC) [4,5]. HSC are precursor mesenchymal cells of elusive origin which characteristically express the intermediate filaments Desmin and Vimentin [4,6]. They are located in the sub-endothelial space between hepatocytes and sinusoidal endothelium, i.e., the space of Disse, and they represent about 10% of all resident cells of the liver. In quiescent state they are non-proliferative and store Vitamin A containing lipid droplets (LD). Once activated, their LDs get depleted and the cells trans-differentiate morphologically into contractile and proliferative myofibroblasts, which contribute majorly to the ECM-production [7]. A crucial marker for activated HSC is the increased expression of α-smooth muscle actin (α-SMA) [2]. Among other cytokines, upregulated TGF-β1 is considered to be the key mediator of HSC activation within the wide fibrosis etiologies [8,9]. Stimulation occurs, either in a paracrine manner through released TGF-β1 of injured hepatocytes or activated macrophages and others cell types, but also in an autocrine manner from activated HSC [10]. Understanding the reversal of activated HSC back into quiescence or better the prevention of activation of HSC in the first place is of great interest towards the development of therapeutic intervention options, in addition to the elimination of inflammation-provoking and damaging triggers.

In this aspect, studies have focused lately to the protein Perilipin 5 (PLIN5). PLIN5, which is a member of the Perilipin family, a group of LD structural proteins, is highly expressed in oxidative tissues, such as the liver and heart, and regulates cellular lipid homeostasis [11,12]. By reducing oxidative stress through inhibiting the lipolysis of LDs, PLIN5 has been shown to have a positive effect in heart and liver injury [13,14]. However, the function of PLIN5 is pleiotropic and varies in a disease-specific and tissue-dependent manner [12,15]. Due to the contrasting consequences of inhibited lipolysis and LD accumulation in the different cell types of the liver, a precise breakdown of the cell-specific role of PLIN5 is required [16]. PLIN5 overexpression in hepatocytes promotes steatosis, while protecting against oxidative stress, its ablation is thought to increase insulin resistance but also to have a protective effect against liver injury [17,18,19,20]. Regarding HSC, it was observed that PLIN5 expression is decreased in activated cells. Exogenous overexpression of PLIN5 in those cells restored LDs and reversed activation. Various mechanisms have been identified. Transcriptional programs of lipogenic gene expression were induced, while pro-lipolytic ones were suppressed. Intracellular oxidative stress was reduced by decreasing reactive oxygen species and elevating glutathione. As the central triggering factor of these mechanisms, the activation of 5′-AMP-activated protein kinase (AMPK) by PLIN5 was identified [21,22]. Previous research showed that activated AMPK also has an indirect attenuating influence on TGF-β1 signaling independent of SMAD2/3 activation [23]. Since PLIN5 seems to play a crucial role in HSC activation and is able to influence signaling pathways and gene transcription, we investigated whether PLIN5 could prevent HSC activation by a direct influence on the pivotal TGF-β1 signal transduction via cell culture experiments with immortalized human and murine HSC lines (i.e., LX-2 and Col-GFP). We observed that overexpression of PLIN5 counteracts TGF-β1-induced activation of HSC in vitro, mediated by attenuation of the SMAD2/3 signaling cascade and furthermore by reduction of STAT3 activity.

## 2. Materials and Methods

### 2.1. Animals for In Vitro and In Vivo Experiments

C57BL/6 wild type (WT) and *Plin5*^−/−^ mice were housed with 3–5 mice/cage. They were maintained at a constant temperature (20 °C) with a relative humidity of 50% and a 12 h of light and 12 h of darkness light cycle. All animals were fed ad libitum a normal chow (V1534, ssniff Spezialdiäten GmbH, Soest, Germany). All animals from which liver tissue was excised or primary cells were isolated were treated in full compliance with the guidelines for animal care and the protocols used were approved by the institutional German Animal Care Committee (LANUV, Recklinghausen, Germany; permit nos.: 84-02.04.2017.A268 and 81-02.04.2020.A228).

### 2.2. Isolation and Culture of Primary HSC

Primary HSC were isolated from 62–69 week old WT and 70–78 week old *Plin5* deficient (*Plin5^−/−^*) male mice [20,24] by pronase and collagenase digestion of liver tissue in situ, density gradient centrifugation, and subsequent flow cytometric purification according to a protocol previously published [25].

Isolated cells were cultured in Dulbecco’s modified Eagle’s medium (DMEM) supplemented with 10% (*v*/*v*) fetal bovine serum (FBS), 4 mM l-glutamine, 100 IU/mL penicillin and 100 µg/mL streptomycin (all from Sigma-Aldrich, Taufkirchen, Germany). The medium was refreshed every second day. On day nine after seeding, when activation and trans-differentiation of the HSC was fully achieved [26,27], the cells and supernatants were harvested for protein expression analyses and Oil Red O staining. 

### 2.3. Cell Lines and Cell Culture

For stimulation experiments with transfected HSC, the cell lines Col-GFP [28] and LX-2 were used [29,30]. Col-GFP are a non-commercial murine HSC cell line that expresses GFP under the control of the C*ol1* promoter/enhancer and it is shown to be a suitable tool for investigating profibrogenic signal transduction of TGF-β1 [28]. LX-2 is an established, genetically well-characterized human HSC cell line with retained key features of HSC. These cells are commonly used for TGF-β1 stimulation and are known for their high transfectability [29,30]. Both cell lines were cultured in DMEM supplemented with 10% (*v/v*) fetal bovine serum (FBS), 2 mM L-glutamine, 100 IU/mL penicillin, 100 µg/mL streptomycin, and 1 mM sodium pyruvate (all from Sigma). In the experiments, transfection was performed first, followed by stimulation with inhibition if required. All experiments and analyses were performed in triplicate.

### 2.4. Plasmid Transfection

The two genes used, *Plin5*, the main target gene under investigation, and *GFP* the negative control (Ctr), were each cloned in the plasmid backbone pCMV-Sport6 from ImaGenes GmbH (Source BioScience, Nottingham, UK). Primary HSC and Col-GFP were transfected with Lipofectamine 2000 Reagent (Thermo Fisher Scientific, Dreieich, Germany) at a DNA to reagent ratio of 2 µg to 4 µL in six well plates according to the manufacturers’ instructions. For Col-GFP experiments, 150,000 cells per well were seeded on the previous day to prepare 70–90% confluent plates. The transfection solution was applied for 6–24 h. The transfection efficiency observed by the fluorescence of the GFP positive cells was 30% for the primary cells and 60% for the Col-GFP cells respectively. LX-2 cells were transfected with TransIT-LT1 Transfection Reagent (Mirus, VWR International GmbH, Darmstadt, Germany) at a DNA to reagent ratio of 2 µg to 6 µL in six well plates according to the manufacturer’s instructions. For LX-2 experiments, 250,000 cells per well were seeded on the previous day to prepare 70–90% confluent plates. The transfection solution was applied for 24 h. Transfection of LX-2 cells was estimated to 60%. In order to distinguish methodological influences from the effects of the target gene *Plin5*, untreated cells, cells treated only with transfection medium (Tf.M.) without vector and cells transfected with the *GFP* vector served as controls. For transfection, Penicillin-streptomycin-free medium was applied 1 h before the transfection. The transfection was performed according to the transfection reagent’s manufacturer.

### 2.5. TGF-β1 Stimulation and Inhibition

For stimulation with TGF-β1 (R&D Systems, Wiesbaden, Germany), HSC were starved in 0.5% (*v/v*) FBS medium for 6–24 h. The medium was further reduced to 0.2% (*v/v*) FBS and the cytokine was added at a concentration of 1 ng/mL for Col-GFP and 2.5 ng/mL for LX-2 and cells stimulated for the indicated time intervals of 30 min, 3 h, 24 h or 48 h. In case of additional inhibition of TGF-β receptor type I (TGFβRI), HSC were pre-treated with 5 µM of the selective TGFβRI (ALK5) inhibitor SB431542 (Tocris Bioscience, Merck, Darmstadt, Germany) for 30 min prior to stimulation. To exclude effects of the inhibitor’s carrier solution (i.e., dimethyl sulfoxide, DMSO), controls were conducted in parallel both with and without DMSO. Subsequently, the cells were harvested accordingly either for Western blot analysis or reverse transcription quantitative real time PCR (RT-qPCR) analysis, as described below.

### 2.6. Western Blot Analysis

For Western blot analysis, mouse livers and HSC were harvested in RIPA-lysis buffer containing protease and phosphatase inhibitors. Protein extraction was performed via sonication followed by centrifugation. After colorimetric protein quantification with DC protein assay (Bio-Rad Laboratories, Düsseldorf, Germany) according to the manufacturers’ instructions, samples of equal protein amount (Col-GFP 40 µg/well, LX-2 20 µg/well, primary HSC 3 µg/well, tissue 100 µg/well) were prepared by adding 1 M dithiothreitol (DTT-1M) and NuPAGE-LDS electrophoresis sample buffer (Thermo Fischer). For cell lysate supernatant analyses, 45 µL of each supernatant was mixed with LDS sample buffer (4×) and DTT-1M. Preparation of samples for Western blot analysis was performed as described previously [20]. Primary and secondary antibodies used are listed in Supplementary Table S1. Visualization was performed using the SuperSignal West Dura Extended Duration Substrate and the myECL TM imager (both from ThermoFisher).

### 2.7. Oil Red O Staining

HSC seeded on glass slides were rinsed with phosphate buffered saline (PBS) and then fixed with 4% paraformaldehyde for 15 min. This was followed by 5 min incubation in 60% isopropanol and then 10 min staining in Oil Red O working solution (Sigma-Aldrich), filtered through two Whatman paper filters. Thereafter, counterstaining with hematoxylin for 5 min (Sigma Aldrich) was performed to stain the nuclei. After fixation and staining, the cells were rinsed thoroughly with distilled water. Finally, they were prepared for microscopy with an aqueous mounting solution (Abcam, Berlin, Germany).

### 2.8. RNA Analysis

Total RNA isolated from HSC was extracted, purified and reverse transcribed using standard protocols as published before [20]. The samples were quantified with the Nanodrop 2000 spectrophotometer (ThermoFisher). The primer pairs used to amplify targeted mRNA via RT-qPCR were designed using the Universal Probe Library tool (Roche, Mannheim, Germany) and are listed in Supplementary Table S2. The targeted mRNAs were amplified in 40 cycles and relative concentrations were calculated using the comparative CT method [31]. All different mRNA were normalized to the mRNA expression of β-actin. The mRNA expression of treated cells was normalized to the mRNA content of the control groups.

### 2.9. Statistical Analysis

For the qRT-PCR results, statistical analyses of expression data were performed using the Student’s test for a comparison of the individual groups. Probability values of *p* ≤ 0.05 were considered statistically significant. Results are expressed as the mean ± standard deviation (SD). All statistical analyses were performed using Excel Analysis 2010 and Windows 2010.

## 3. Results

### 3.1. The Crucial Role of Plin5 in HSC Activation

#### 3.1.1. *Plin5* Deficiency Promotes Extracellular Matrix Protein and Mesenchymal Marker Expression In Vivo and In Vitro

**Figure 1:**
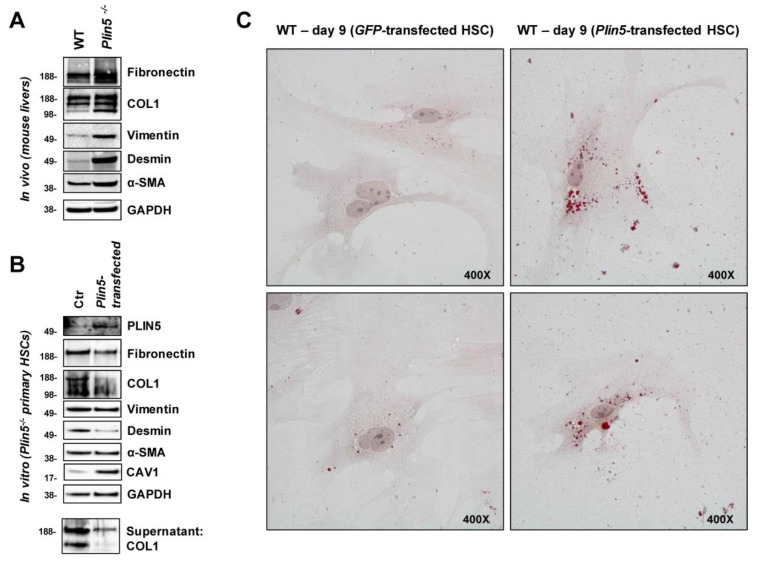
The impact of PLIN5 on hepatic fibrogenesis in mice in vivo and on primary mouse HSC in vitro. (**A**) Western blot analysis of proteins extracted from whole liver of 35 weeks old wild type (WT) and *Plin5^−/−^* mice (*n* = 4–5 mice per experimental group), while exemplary results from each one mouse are shown. (**B**) Western Blot analysis of cell lysates and supernatants of activated primary HSC from 70–78 weeks old *Plin5^−/−^* mice cultured for 9 days and transfected with an expression construct for *Plin5* on day 8. (**C**) Oil Red O staining of activated primary HSC from 62–69 weeks old WT mice cultured for 9 days and transfected with an expression construct for *Plin5* or green fluorescent protein (GFP) constructs on day 8. Microscope images were taken at 400× magnification. *Plin5*^−/−^, *Plin5* deficient; Ctr, control.

Our in vivo study on mouse livers gave rise to the assumption of increased fibrogenic activity due to the ablation of *Plin5*. The Western blot analysis of protein extracted from whole livers of around 35 weeks old WT and *Plin5^−/−^* male mice, fed with normal chow, shows a clear enhancement of the ECM proteins Fibronectin and COL1 as well as an enhancement of α-SMA in *Plin5^−/−^* mice compared to WT. The mesenchymal markers Vimentin and Desmin are highly expressed in *Plin5*^−/−^, whereas they are almost undetectable in the liver of WT mice (Figure 1A). This finding could suggest a protective role of PLIN5 against increased mesenchymal activity with the augmented production of ECM proteins, which requires an HSC-specific approach as HSC play a major role during hepatic fibrogenesis.

We then particularly examined primary HSC isolated from 70 to 78 weeks old male *Plin5^−/−^* mice and tested whether the increased activation, observed in vivo, could be replicated and further reversed by overexpression of exogenous PLIN5. The isolated primary HSC were cultured for nine days before being harvested for analysis. On the eighth day, they were transfected with a *Plin5*-construct. The detection of PLIN5 protein overexpression confirmed the transfection. ECM proteins and mesenchymal markers as well as α-SMA were strongly expressed in *Plin5^−/−^* Ctr cells. The PLIN5 overexpression after transfection caused a reduction of Fibronectin and COL1 as well as Desmin. There was also a reduced amount of COL1 detected in the supernatant due to the exogenous PLIN5. Only Vimentin and α-SMA showed no alterations in expression (Figure 1B). The observations confirm the assumption that lack of PLIN5 leads to the activation of HSC with high production of ECM, while a quick activation reverse was achieved after overexpression of PLIN5.

Moreover, an altered expression of Caveolin-1 (CAV1) was apparent. CAV1 is considered an inhibitory regulator of TGF-β1 signaling and fibrogenesis in HSC [32,33]. While CAV1 was weakly expressed in *Plin5^−/−^* control cells, its expression was significantly increased by exogenous PLIN5 (Figure 1B). This raises the question whether PLIN5 indirectly interferes with TGF-β1 via CAV1 expression. However, in subsequent cell line experiments, this finding could not be further analyzed due to strong basal expression of CAV1 across the different conditions (data not shown).

#### 3.1.2. PLIN5 Overexpression Provokes Phenotypic Changes in Activated Primary HSC In Vitro

Corresponding to HSC activation due to the absence of PLIN5, it has been shown in return that overexpression inhibits HSC activation [21]. The quiescent HSC phenotype is generally associated with LD accumulation. Using Oil Red O staining, we were able to show the restoration of LDs in activated primary HSC by overexpression of PLIN5, indicating that PLIN5 contributes to HSC activation reverse. Isolated primary HSC from 62–69 weeks old male WT mice were cultured for nine days before staining. On the eighth day, the cells were transfected with either a *Plin5*- or *Gfp*-expression construct. When examined under the microscope, at 400× magnification, there was distinctly higher amount of small red-stained LDs in the cells which overexpressed PLIN5 compared to the control one (Figure 1C). This observation demonstrates a phenotypic change towards quiescent HSC due to overexpression of PLIN5.

#### 3.1.3. Enhanced PLIN5 Expression Diminishes TGF-β1 Induced Extracellular Matrix Protein Expression, Hepatic Stellate Cell Activation, and Protected against Fibrogenesis in Col-GFP and LX-2 Cells

**Figure 2:**
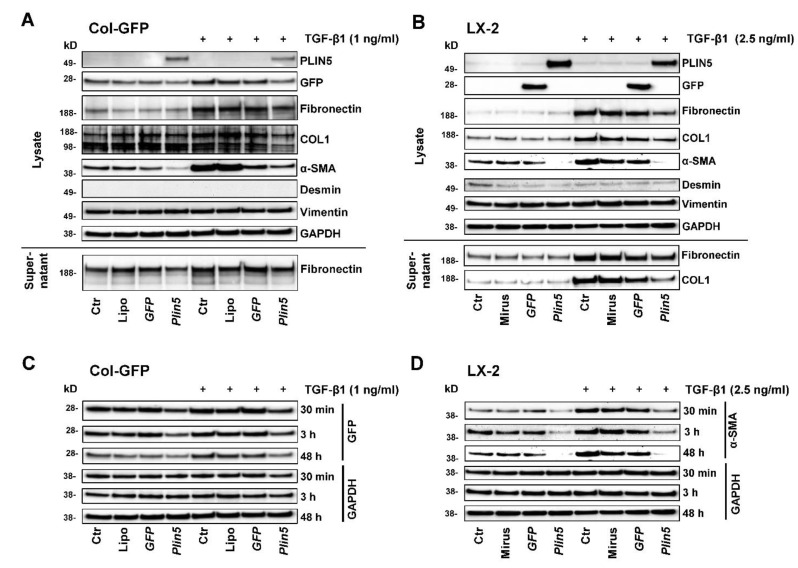
Effects of *Plin5* overexpression on HSC activation mediated by TGF-β1 stimulation in vitro. (**A**,**B**) Western blot analysis of *Plin5* transfected Col-GFP and LX-2 cells and control conditions, stimulated with TGF-β1 for 48 h where indicated. Negative controls to *Plin5* transfection include an untreated control, a transfection medium-only treated control and a GFP-transfected control. The expression of GFP and PLIN5 as transfection proof, expression of extracellular matrix proteins and mesenchymal markers of cell lysates, as well as Fibronectin and COL1 expression/secretion of the supernatants are shown. The experiments were performed and evaluated in triplicate. (**C**) GFP expressions in Col-GFP cells with and without stimulation over different time intervals (30 min, 3 h, and 48 h) are demonstrated separately. (**D**) α-SMA expression in LX-2 with and without TGF-β1 stimulation over different time intervals (30 min, 3 h, and 48 h). All experiments were performed in triplicate. Lipo, Lipofectamin; Ctr, control.

To investigate an intervention of PLIN5 on TGF-β1-induced HSC activation, we performed stimulation and transfection experiments with two cell lines, murine Col-GFP-HSC and human LX-2. Firstly, we monitored their response to TGF-β1 stimulation and examined whether overexpression of PLIN5 had a protective effect. For each cell line, TGF-β1 was applied on *Plin5*-transfected cells as well as the respective controls and they were compared to the unstimulated equivalents. Additionally, controls treated with transfection reagent only and GFP transfected controls were implemented to be able to differentiate methodological implications. To ensure successful transfection, the protein expression of the transfected genes was analyzed, which showed the expected overexpression (Figure 2A,B). LX-2 cells revealed higher transfection efficiency than the Col-GFP HSC. The detection of GFP-transfection in Col-GFP is superposed by the intrinsic GFP expression. The transfection seems to reveal stronger expression of the proteins of interest at earlier time points (Figure 2C). In both cell lines, an upregulation of HSC activity through TGF-β1 stimulation was shown by the increased expression of COL1, Fibronectin, and α-SMA after 48 h of TGF-β1 stimulation (Figure 2A,B). The basal low expression of Fibronectin and COL1 in the non-treated with TGF-β1 cells revealed no difference in their expression after PLIN5 overexpression. Their expression appeared to be strongly upregulated by TGF-β1 stimulation which was further negatively affected after PLIN5 overexpression. On the other hand, α-SMA, which was already expressed in the non-stimulated cells, showed significant downregulation after PLIN5 overexpression. This basal activation shown by α-SMA expression is a common characteristic of these cell lines and also visible at early times (Figure 2D) [28,29,30]. In contrast to long-term TGF-β1 stimulation-induced expression of ECM-proteins, TGF-β1 enhanced cell activation, as measured by α-SMA, even after short durations of stimulation (Figure 2C,D). Col-GFP cells, which express GFP under the control of the *collagen (I)* promoter/enhancer also showed their intrinsic activation through this specific expression of GFP (Figure 2C). In contrast to the original characterization of this cell line, which reported no enhanced GFP expression after TGF-β1 stimulation for 4 h [28], a slight effect on expression of GFP appears to be visible at 48 h (Figure 2A,C). The supernatant also showed higher levels of secreted Fibronectin and COL1 (COL1 was only detectable in LX-2 supernatant) in response to TGF-β1 stimulation. Vimentin was expressed by both cell lines, whereas Desmin was only faintly detectable. However, neither protein showed any alterations under stimulation with TGF-β1 (Figure 2A,B). Overexpressed PLIN5 reduced the basal activity of unstimulated cells, which is visible in the expression of α-SMA in both cell lines and GFP-expression in Col-GFP at all times (Figure 2). In conclusion, both cell lines responded to TGF-β1, in particular by induced ECM protein expression (i.e., COL1 and Fibronectin) as well as augmented α-SMA expression. The protective effect of overexpressed PLIN5 was strong also after stimulation with this cytokine.

### 3.2. Non-Canonical (MAPK) Pathways Are Unaffected of TGF-β1-Induced HSC-Activation and PLIN5 Overexpression

Besides the main axis of TGF-β1 signal transduction, the SMADs, there are a number of noncanonical pathways, all of which interact with each other. We examined effects on nuclear factor-κB (NF-κB) and the group of mitogen-activated protein kinases (MAPKs), to which specific roles in hepatic fibrogenesis, HSC activation, and TGF-β1 function are attributed.

**Figure 3:**
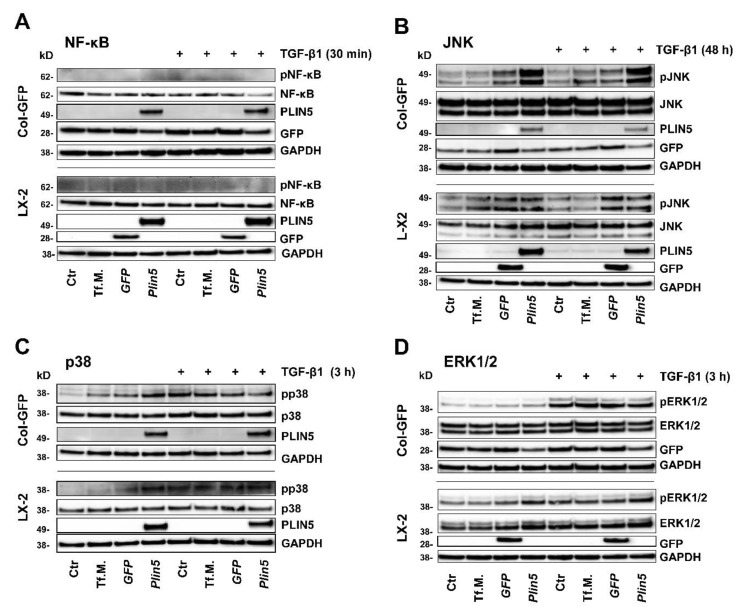
Unclear involvement of MAPKs and NF-κB in TGF-β1-mediated activation of HSC-cell lines and no or indistinct effect of *Plin5*-transfection. Western blot analysis of protein extracts isolated from *Plin5* transfected Col-GFP and LX-2 cells stimulated with TGF-β1 for indicated time intervals (Col-GFP, 1 ng/mL; LX-2, 2.5 ng/mL). (**A**) Phosphorylated and total NF-κB quantities 30 min after TGF-β1 stimulation were determined. (**B**) Phosphorylated and total JNK after stimulation with TGF-β1 for 48 h. (**C**) Phosphorylated and total p38 after 3 h stimulation. (**D**) Phosphorylated and total ERK1/2 after 3 h stimulation. All experiments were performed in triplicate. Tf.M., transfection medium; Ctr, control.

Activation of the transcription factor NF-κB in HSC is assumed to be a key mediator of fibrosis by having an activating and survival effect via multiple mechanisms [34]. In our in vitro studies, an immediate activation of NF-κB through phosphorylation of Ser536 after TGF-β1 stimulation for 30 min (nor later, data not shown) could not be detected. Moreover, NF-κB expression seemed independent to PLIN5 overexpression because total NF-κB was uniformly expressed in both cell lines across the chosen conditions (Figure 3A).

The extracellular signal-regulated kinase (ERK) signaling pathway is presumed to play a prominent role in TGF-β1 signal transduction, although there is also some indication of suppressive properties [35,36]. Our investigations revealed a strong activation of the ERK signaling pathway in Col-GFP after 3 h of TGF-β1 stimulation, without an influence of PLIN5 overexpression (Figure 3D). Although in LX-2, TGF-β1 stimulation had a negligible effect, the cells tended to show a stronger phosphorylation of ERK in PLIN5 overexpression after 30 min (data not shown) and 3 h of TGF-β1 stimulation (Figure 3D). The results of the ERK investigations showed a discrepancy between the cell lines, allowing for conflicting interpretations.

The MAPKs p38 and JNK were investigated 30 min, 3 h and 48 h after TGF-β1 stimulation. The kinase p38 showed consistent, slight, and indistinct phosphorylation without clear influence of TGF-β1 or PLIN5 overexpression (exemplarily shown 3 h stimulation in Figure 3C). JNK phosphorylation was similarly unaffected and without a clear coherent effect of TGF-β1 stimulation or PLIN5 overexpression at 30 min and 3 h (results not shown). Yet, the 48 h samples showed TGF-β1-independent phosphorylation after application of transfection medium (Tf.M.), as well as in cells transfected with *Gfp-* and *Plin5*-expression constructs, with increasing intensity and focus on PLIN5 overexpression (Figure 3B). However, it is not possible to clearly determine a late effect of PLIN5 overexpression, as this could have been provoked by cellular stress caused by the transfection.

### 3.3. PLIN5 Overexpression Attenuates TGF-β1-Stimulated HSC Activation via SMAD Signaling

**Figure 4:**
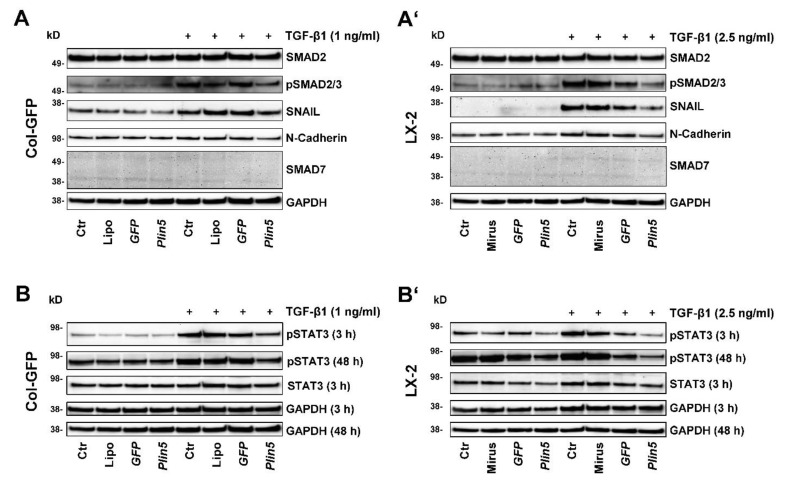
PLIN5 overexpression attenuates TGF-β1 signal transduction and downstream target expression via SMAD2/3 signaling pathway and further inhibits the activation of STAT3. Western blot analysis of *Plin5* transfected Col-GFP and LX-2 cells stimulated with TGF-β1 for indicated time intervals (Col-GFP, 1 ng/mL; LX-2, 2.5 ng/mL). (**A**,**A’**) show the expression of phosphorylated SMAD2/3 and total SMAD2 after 48 h stimulation, as well as the expression of the downstream targets SNAIL, N-Cadherin, and SMAD7. (**B**,**B’**) depict the phosphorylation of STAT3 at Tyr705 after stimulation with TGF-β for 3 h and 48 h and unstimulated and total STAT3 after 3 h stimulation. All experiments were performed in triplicate. Ctr, control; Lipo, Lipofectamin.

The SMAD signaling pathway, known as a pivotal intracellular effector pathway for TGF-β1, was strongly, early, and persistently activated by stimulation in our cell culture experiments in both cell lines, Col-GFP and LX-2. Phosphorylation of SMAD2/3 was detectable after TGF-β1 stimulation (Figure 4). The overexpression of PLIN5 had a strong attenuating effect on SMAD2/3 activation (Figure 4A,A’). This effect also extended to the downstream targets of this pathway. SNAIL expression, a transcription factor activated by SMAD2/3 signaling, was promoted by TGF-β1 stimulation, but significantly reduced by PLIN5 overexpression (Figure 4A,A’). The expression of neuronal cadherin (N-cadherin), a transmembrane glycoprotein that subordinates the transcription factor SNAIL and is characteristic for activated HSC, again showed this correlation. Based on the coherence of the results and the concordance between the two cell lines, we concluded that PLIN5 inhibits partially the activating effect of TGF-β1 on HSC by attenuating the SMAD2/3 pathway.

**Figure 5:**
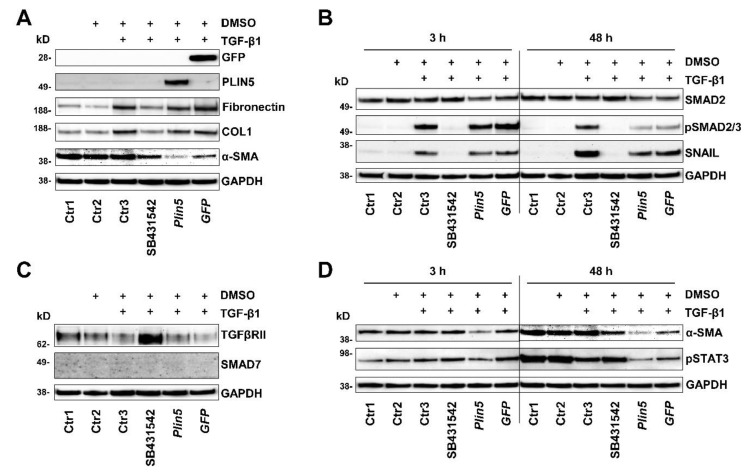
PLIN5 overexpression protects against TGF-β1-mediated STAT3 phosphorylation. Western blot analysis of *Plin5* transfected LX-2 cells compared to TGF-β receptor inhibitor treated cells and control conditions. Cells were stimulated with TGF-β1 (2.5 ng/mL) for indicated time intervals. *GFP* transfection served as a negative control. As a further control, cells that were treated with DMSO serving as the inhibitor carrier medium were analyzed. In addition, three further controls, one untreated (Ctr1), one only exposed to DMSO (Ctr2), and one stimulated and exposed to DMSO (Ctr3) were analysed. (**A**) Analysis of expression of extracellular matrix proteins, GFP, and PLIN5 under these conditions stimulated for 48 h. (**B**) Analysis of SMAD2/3 activation and downstream transcription factor SNAIL. (**C**) Alterations in the expression of the TGFβRII and the SMAD7 after 48 h stimulation are shown. (**D**) Phosphorylation of STAT3 at Tyr705 and expression of α-SMA after TGF-β1 stimulation for 3 h or 48 h. All experiments were done in triplicate.

An inhibition experiment with the inhibitor SB431542 was designed for the investigation of the TGF-β receptor inhibition. Various controls were included to distinguish effects from the carrier solution of the inhibitor, DMSO, stimulation, and plasmid transfection. After a cell treatment of 48 h, a slight decrease of TGF-β receptor type II (TGFβRII) was noticeable in the TGF-β1 stimulated group compared to the unstimulated controls (Figure 5C). Inhibition of TGF-β1 had a small suppressing effect on α-SMA. Induced by TGF-β1, COL1 and Fibronectin expression, on the other hand, was clearly inhibited by the TGFβRI inhibitor, which declares TGF-β1 stimulation mainly as an ECM driver (Figure 5A).

**Figure 6:**
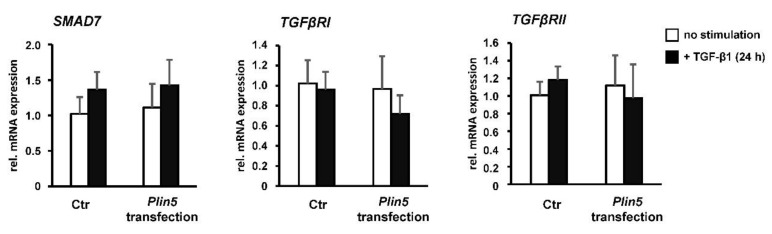
PLIN5 overexpression and TGF-β1 stimulation have no significant effect on inhibitory *Smad7*. *Plin5*- transfected LX-2 cells stimulated with TGF-β1 (2.5 ng/mL) for 24 h or left unstimulated. All experiments were performed in triplicate.

The aforementioned phosphorylation of SMAD2/3 was completely prevented by treatment with the TGFβRI inhibitor (Figure 5B), while PLIN5 overexpression obstructed SMAD2/3 phosphorylation. In search for possible causes of SMAD2/3 attenuation, we investigated alterations in the expression of the inhibitory SMAD7 and the TGFβRII. There was no evidence for a possible increased expression of the inhibitory SMAD7 by PLIN5 overexpression, neither on protein expression nor on RNA level analyzed by RT-qPCR (Figure 4A,A’, Figure 5C and Figure 6).

Reflecting the autoregulatory feedback, a slight increase in *Smad7* transcription by TGF-β1 stimulation was seen in both the Ctrl and *Plin5*-transfected cells, which is, however, not statistically significant (Figure 6). A clearly increased expression of TGFβRII was observed as a result of its inhibition. However, the overexpression of PLIN5 did not mimic this effect to an altered expression of TGFβRII (Figure 5C). In addition, there was no effect detectable on the expression of TGFβRII and TGFβRI at transcriptional level by overexpression of PLIN5 or stimulation (Figure 6). It can therefore be assumed that SMAD signaling attenuation by PLIN5 overexpression is mediated neither by increased inhibitory SMAD7 nor by inhibition of the TGFβRI.

### 3.4. Exogenous PLIN5 Prevents STAT3 Phosphorylation

In our study, we investigated the JAK-STAT pathway and observed the phosphorylation of STAT3 after PLIN5 overexpression. The HSC showed basal activation of STAT3, clearly visible after 48 h (Figure 4B,B’), which seemed to increase over time (Figure 5D). TGF-β1 stimulation tended to lead to increased phosphorylation of STAT3. This is more evident in Col-GFP versus LX-2 and in the early 3 h versus 48 h stimulation. After 48 h, phosphorylation was detectable at comparable levels across the groups (Figure 4B,B’). In the inhibition experiment, TGF-β1 stimulation showed lower phosphorylation of STAT3 after 48 h compared to the unstimulated controls. The TGFβRI inhibitor SB431542 was able to antagonize this reduced expression (Figure 5D). PLIN5 overexpression clearly inhibited TGF-β1 induced STAT3 activation in both cell lines (Figure 4B,B’). The inhibition experiment further revealed that blocking the TGFβRI surprisingly did not prevent the phosphorylation of STAT3, indicating that it might be mediated in ways other than TGFβRI (Figure 5D). STAT3 phosphorylation bears resemblance to the expression of α-SMA, which was likewise strongly reduced by PLIN5 overexpression, but only slightly by the TGFβRI inhibitor. The results show an early TGF-β1-induced STAT3 activation (30 min) as well as an increasing and independent phosphorylation of STAT3 over time. PLIN5 seems to exert a strong inhibitory influence on STAT3 phosphorylation, independent of the activation genesis.

## 4. Discussion

Activated HSC represent the major contributor to ECM accumulation and therefore play a pivotal role in the pathophysiology of hepatic fibrogenesis [4,5]. Underlying mechanisms and their triggers are already well explored [7], while the cytokine TGF-β1 has already been identified as a key mediator in fibrogenesis [8,9]. However, tools for the specific suppression of activation and therefore hepatic protection are not yet fully developed and remain the subject of research. PLIN5, a structural LD protein highly expressed in oxidative tissues, has been identified to have an inhibitory effect on HSC activation. The mechanisms known so far are mainly related to fat metabolism processes [21,22]. Therefore, in our study, we investigated the interaction of PLIN5 and TGF-β1 using appropriate in vitro experiments with HSC-Col and LX-2 cells and discovered a clear intervention in signal transduction.

First, we were able to demonstrate the important role of PLIN5 in livers and primary HSC of mice. Our in vivo experiments showed increased ECM protein and mesenchymal marker expression in the liver of *Plin5^−/−^* mice compared to WT mice (Figure 1). Via our recent animal studies, we showed that *Plin5^−/−^* mice in a 30-week high-fat diet did not have higher hepatic injury in histological studies compared to WT, but surprisingly less [20]. The reason for this could be a cell-specific role of PLIN5 in the context of the fat paradox [16]. Moreover, the 30-week high fat diet did not lead to fibrotic development [20]. Consequently, our previous study in companion with the current project suggests that PLIN5 has a pleiotropic role in distinct stages of liver damage from early inflammation to steatohepatitis and later progress towards fibrosis. These studies require a closer look at enhanced mesenchymal activity through the lack of PLIN5 in a cell-specific approach focused to clarify the importance of PLIN5 in HSC functions.

In the subsequent in vitro investigation, primary HSC isolated from *Plin5^−/−^* mice reflected increased HSC activation, followed by the observation of a decrease in activity after overexpression of PLIN5. Furthermore, we were able to confirm the previously described phenotypic regression of activated primary HSC from WT mice towards a quiescent status by the restoration of LDs through exogenous PLIN5 using Oil Red O staining [21].

A striking aspect of the primary cell study was that CAV1, which was only slightly expressed in *Plin5^−/−^* mice, was significantly increased by exogenous PLIN5. CAV1 is considered an inhibitory regulator of TGF-β1 signaling and fibrogenesis in various organs [32]. Lu et al. showed that *Cav1* deficient^−^ mice subjected to liver fibrosis induced by carbon tetrachloride exhibited enhanced TGF-β1 signaling, and in this context had increased inflammatory injury in comparison to WT mice [33]. These findings made us think that PLIN5 might have a suppressive role against TGF-β1 signaling.

To explore the relationship between PLIN5 and TGF-β1, we performed in vitro cell experiments with a murine (Col-GFP) and a human (LX-2) HSC line. HSC consistently showed an activating response to TGF-β1, with TGF-β1 acting primarily as an ECM driver of these basally activated cells by significantly increasing COL1 and Fibronectin expression. The already expressed α-SMA, a major marker of HSC activation, was also enhanced. PLIN5 overexpression was able to downregulate basal activity as measured by the expression of α-SMA. The clear superiority in this respect compared to the TGFβRI inhibitor, which had hardly any effect (Figure 5), indicates protective features of PLIN5. These circumstances correspond to the previous published works on TGF-β1 in HSC activation and the function of PLIN5 in activated HSC [8,9,21,22]. As a new finding in this context, we were able to show that PLIN5 was capable of explicitly counteracting the TGF-β1-induced alterations in COL1, Fibronectin and α-SMA expression.

The molecular mechanisms of ECM protein induction and HSC activation by TGF-β1 are not yet fully ascertained. It is known that TGF-β1 acts both via its canonical SMAD pathway and the non-canonical MAPKs, which all interact with each other. While the importance of SMAD signaling is evident regarding this process, there is ambivalence about the MAPKs [37]. Although members of the MAPKs such as p38 and JNK have been associated in other studies with HSC activation by TGF-β1 [38,39,40], the same members did not reveal a clear response to TGF-β1 stimulation or an effect after PLIN5 overexpression in our in vitro experiments (Figure 3), which agrees with a previous study by Mu and colleagues [41].

Behaving similarly, the transcription factor NF-κB, which is suggested to play an important role in HSC activation amongst others, also interacts with TGF-β1 [34]. NF-κB was uniformly expressed in the experiments and activation by phosphorylation was not detectable in any condition (Figure 3).

Among the MAPKs, only ERK showed an influence in our in vitro studies, although conflicting. The signaling pathway in Col-GFP cells was induced by TGF-β1 but left uninfluenced by PLIN5. However, in LX-2 cells, ERK got activated by PLIN5 overexpression, while a negligible effect after TGF-β1 stimulation was observed (Figure 3). The former finding is consistent with the findings of ERK, being a prominent signaling pathway of TGF-β1 in relation to hepatic myofibroblast activation and trans-differentiation [35]. As there is also an indication of ERK having a suppressive effect on SMAD signal transduction in murine epithelial mammary cells [36,42], we hypothesize that PLIN5 driven ERK activation might have a protective effect in LX-2. Moreover, PLIN5 was recently shown to abrogate oxidative damage in pancreatic β-cells via an activated ERK signaling pathway, which in turn confirms an activating property of PLIN5 [43]. However, due to the inconsistency between the two cell lines, no general conclusion can be drawn, and further research is required.

The canonical axis SMAD2/3 is undoubtedly considered as a mediator of TGF-β1 induced ECM expression. The underlying mechanism after the binding of TGF-β1 to TGFβRII, following hetero-dimerization of TGFβRII and TGFβRI and subsequent phosphorylation of SMAD2 and SMAD3, which heterocomplex with SMAD4 and further translocate into the nucleus to regulate transcription of multiple target genes, particularly COL1, is already well explored [37,44]. SMAD3 is considered to play an important profibrogenic role in this context whereas SMAD2 has anti-fibrotic properties, but they function cooperatively. SMAD7 on the other hand is an inhibitory SMAD that exerts important auto-regulatory negative feedback on SMAD2/3 signaling during stimulation [9,37]. In both cell lines, we could consistently show that the TGF-β1-SMAD2/3 axis was strongly activated upon stimulation with the cytokine while attenuated by a following PLIN5 overexpression (Figure 4). When investigating the possible mechanisms of PLIN5´s intervention, an increase in inhibitory SMAD7 could not be detected. In addition, PLIN5 did not appear to act as a TGF-β receptor antagonist. The attenuating effect of PLIN5 overexpression also extended to further signal transduction. HSC activation is discussed as an epithelial-mesenchymal-transition (EMT)-like trans-differentiation. Although they are not epithelial but precursor mesenchymal cells with both epithelial and mesenchymal characteristics, their activation shows EMT similarities in several studies [45], such as the switch from E-Cadherin to *N*-Cadherin during HSC activation [46]. Our results support this assumption due to the fact that the EMT-transcription factor SNAIL and downstream target genes such as *N*-Cadherin were increasingly expressed by TGF-β1 stimulation, which is typical for EMT [45,47]. These TGF-β1 mediated changes were also mitigated by PLIN5 overexpression (Figure 4).

In summary, PLIN5 reduced in vitro the TGF-β1-induced ECM protein expression of HSC by attenuating SMAD2/3 signaling, as well as their EMT-like trans-differentiation by attenuating the SMAD2/3 downstream targets SNAIL and *N*-cadherin.

Recent research provides strong evidence that, in addition to SMAD signaling, STAT3 also plays a significant role in TGF-β1-mediated HSC activation. In response to TGF-β1, STAT3 appears to be directly phosphorylated by JAK1, as a constitutive TGFβRI binding protein, in a SMAD-independent manner, which then leads to the increased expression of COL1 and α-SMA in addition to and in cooperation with SMAD3 [48,49]. Other studies reveal a more reciprocal relationship between TGF-β1 and STAT3 and also showed increased TGF-β1 expression by alternatively activated STAT3, such as through Interleukin 6 (IL-6) [50]. Inhibitory approaches to STAT3 as a therapeutic option showed reduced HSC activation and ECM expression, as well as reduced phosphorylation of SMAD2/3, which again suggests crosslinking of the two pathways in both directions [51,52]. In our experiments, the cells already showed a basal activation of STAT3 in the control groups, which can be explained by the already semi-activated state of HSC lines when in cell culture, which increased highly after TGF-β1 stimulation over time. Activation induced by TGF-β1 was particularly evident after 3 h stimulation and was still strongly induced after 48 h (Figure 4).

In our study, the overexpression of PLIN5 was clearly protective against STAT3 activation. The expression resemblance between phosphorylated STAT3 and α-SMA is remarkable: both showed basal expression in the control cells as well as increased expression after TGF-β1 stimulation while a strong reduction in expression when PLIN5 overexpression followed (Figure 4). Moreover, α-SMA was only slightly reduced by TGF-β1 inhibition (Figure 5), indicating that the reduced phosphorylation of STAT3 by PLIN5 overexpression is the main cause for the lower activation level of HSC.

Another link between STAT3 and TGF-β1 is described by Wang et al., who identified STAT3 as a positive regulator of TGF-β1-induced-EMT in hepatocytes. Inhibition of its phosphorylation led to an attenuation of the SNAIL-SMAD3/TGF-β1 signaling pathway [53]. Moreover, a direct influence of activated STAT3 on the EMT transcription factor SNAIL is described in various cancer cell types [54]. Such a supportive effect would also be potential in the EMT-like trans-differentiation of HSC, observed in our experiments.

In sum, the results suggest that PLIN5 suppresses STAT3 phosphorylation in basal or TGF-β1 stimulation conditions. Down-regulated STAT3 results in lower ECM proteins as well as α-SMA expression. Considering the results of the STAT3 inhibition experiments, the previously described TGF-β1-SMAD2/3 attenuation by PLIN5 overexpression could be mediated or is at least supported by STAT3. Nevertheless, the exact interrelation between the different pathways and mediators remains unclear.

## 5. Conclusions

It can be concluded that our in vivo and in vitro results demonstrated the importance of PLIN5 in preventing HSC activation and ECM production, especially against TGF-β1 stimulation, thus serving as a potential protection against hepatic fibrogenesis. This was mediated through the attenuation of TGF-β1 signaling pathway SMAD2/3 and downstream SNAIL, decreasing both induced ECM production and EMT-like trans-differentiation of HSC as its target genes. In addition, PLIN5 caused a suppression of STAT3 activity, which has been shown to have an alleviating effect on activated HSC through multiple mechanisms such as TGF-β1 treatment. We therefore here suggest that that down-regulated STAT3 seems to be a possible mediator of TGF-β1-SMAD2/3 attenuation by PLIN5. Alongside the already known protective mechanisms of PLIN5, these new insights derived from our studies reveal an even more versatile and promising role of PLIN5 in attenuating HSC activation and liver fibrogenesis. Increasing numbers of studies investigating the roles of PLIN5 in inflammation, lipid metabolism, and fibrogenesis brought this protein into the focus of clinical hepatology. However, more research on the pleiotropic functions is necessary to clarify the types of liver conditions in which PLIN5 could serve as a therapeutic target.

## Data Availability

All data analyzed during this study are included in this published article (and its supplementary information files). Specific requests can be made via E-mail to the corresponding authors.

## Supplementary Materials

The following are available online at https://www.mdpi.com/article/10.3390/cells10092184/s1, Table S1: Antibodies used for Western blot analysis, Table S2: Oligonucleotides used for quantitative real time PCR.

## Abbreviations

α-SMA	α-smooth muscle actin
AMPK	5′-AMP-activated protein kinase
CAV1	Caveolin1
COL1	collagen type I
Ctr	control
DMEM	Dulbecco modified Eagle medium
DMSO	dimethyl sulfoxide
DTT	dithiothreitol
ECM	extracellular matrix
EKR	extracellular signal-regulated kinase
EMT	epithelial-mesenchymal-transition
FBS	fetal bovine serum
h	hours
HSC(s)	hepatic stellate cell(s)
JNK	c-Jun *N*-terminal kinases
LDs	lipid droplet(s)
MAPK	Mitogen-activated protein kinases
min	minute(s)
N-Cadherin	neuronal Cadherin
NF-κB	Nuclear factor-κB
p38	p38 mitogen-activated protein kinases
PBS	phosphate-buffered saline
PLIN5	Perilipin 5
RT-qPCR	reverse transcription quantitative real time PCR
SD	standard deviation
STAT3	signal transducers and activators of transcription 3
TBST	Tris-buffered saline
Tf.M.:	transfection medium
TGF-β1	transforming growth factor-β1
TGFβR	TGF-β receptor
WT	wild type
