# Supplementary material for: Perilipin 5 Ameliorates Hepatic Stellate Cell Activation via SMAD2/3 and SNAIL Signaling Pathways and Suppresses STAT3 Activation
Source: Cells. 2021 Aug 24;10(9):2184. doi: 10.3390/cells10092184 (PMC8467115; doi:10.3390/cells10092184)
Supplement: Supplementary file 1 [file cells-10-02184-s001.zip › Table S1.pdf]

Table S1. Antibodies used for Western blot analysis

Primary antibodies					
**Antibody**	**Cat. No.**	**Clonality**	**Supplier**	**Reactivity**	**Dilution**
Caveolin 1	#3238	poly - rabbit	Cell Signaling	h,m,r,z,hm	1:1,000
Collagen 1	ab34710	poly - rabbit	Abcam	h,m	1:1,000
Desmin	D93F5	mono-rabbit	Cell Signaling	h,m,r	1:1,000
Vimentin	Ab92547	mono - rabbit	Abcam	h,m,r	1:1,000
Fibronectin	AB1954	poly - rabbit	Millipore-Merck	h,m,r	1:1,000
α-SMA	CBL171	mono - mouse	Millipore	h,m,r,e,c	1:1,000
GAPDH	sc-32233	mono - mouse	Santa Cruz	h,m,r	1:1,000
GFP	sc-8334	poly - rabbit	Santa Cruz	GFP	1:500
PLIN5	GP31	poly - guinea pig	ProGen	h,m,r	1:1,000
NF-κB	sc-8008	mono - mouse	Santa Cruz	h,m,r	1:1,000
pNF-κB	#3031	rabbit	Cell Signaling	h,m,r,mk	1:1,000
p38	#9228	Mouse IgG1	Cell Signaling	h,m,r,mk,pg,sc,	1:1,000
pp38	BD612281	Mouse IgG1	BD Bioscience	h,m,r,	1:1,000
ERK1/2	#9102	rabbit	Cell signaling	h,m,r,hm,mk, mi,dm,z,b,pg,ce,	1:1,000
pERK1/2	#9101	rabbit	Cell Signaling	h,m,r,hm,mk, mi,dm,z,b,pg,ce	1:1,000
JNK	#9252L	rabbit	Cell Signaling	h,m,r,hm,mk,z,b,sc	1:1,000
pJNK	#9251S	rabbit	Cell Signaling	h,m,r,hm,mk,dm,b,sc	1:1,000
SMAD2	#3103	Mouse IgG1	Cell Signaling	h,m,r,mk	1:1,000
pSMAD2/3	#8828	mono-rabbit	Cell Signaling	h,m,r,mk	1:1,000
SNAIL	#3879	mono-rabbit	Cell Signaling	h,m,r,mk	1:1,000
STAT3	sc-7179	rabbit	Santa Cruz	h,m,r	1:1.000
pSTAT3 (Tyr)	#9145	rabbit	Cell Signaling	h,m,r,mk	1:2,000
TGFβRII	#79424	rabbit	Cell Signaling	h,m,r	1:1,000
SMAD7	sc-365846	mono-mouse	Santa Cruz	h,m,r	1:1,000
N-Cadherin	ab98952	mono - mouse	Abcam	h,m,r	1:1000
**Secondary antibodies**					
**Antibody**	**Cat. No.**	**Clonality***	**Supplier**	**Reactivity**	**Dilution**
goat anti-mouse IgG-HRP	sc-2005	poly	Santa Cruz	m	1:10,000
goat anti-rabbit IgG-HRP	31460	poly	Thermo Scientific	rb	1:10,000
goat anti-guinea pig IgG-HRP	AP108P	poly	Millipore-Merck	gp	1:10,000

* Abbreviations used are: mono, monoclonal antibody; poly, polyclonal antibody; h, human; m, mouse; r, rat; rb, rabbit; g, goat; gp, guinea pig; hm, hamster; mk, monkey; mi, mink; c, chicken; dm, *D. melanogaster*; x, Xenopus; z, zebrafish; b, bovine; dg, dog; pg, pig; e, equine; ce, *C. elegans*; sc = *S. cerevisiae*.
