# Supplementary material for: Perilipin 5 Ameliorates Hepatic Stellate Cell Activation via SMAD2/3 and SNAIL Signaling Pathways and Suppresses STAT3 Activation
Source: Cells. 2021 Aug 24;10(9):2184. doi: 10.3390/cells10092184 (PMC8467115; doi:10.3390/cells10092184)
Supplement: Supplementary file 1 [file cells-10-02184-s001.zip › Table S2.pdf]

Table S2. Oligonucleotides used for quantitative real time PCR

Human Gene	Accession No.	Primers
Smad7	NM_005904.3	forward: 5′-agccgactctgcgaactaga-3′
		reverse: 5′-attcgttcccccctgtttca-3′
TGFβRI	NM_004612.3	forward: 5′-aaattgctcgacgatgttcc-3′
		reverse: 5′-cataataaggcagttggtaatcttca-3′
TGFβRII	NM_001024847.2	forward: 5′-caccgcacgttcagaagtc-3′
		reverse: 5′-tggatgggcagtcctattaca-3′
β-actin	HQ154074.1	forward: 5′-ccacgaaactaccttcaactcc-3′
		reverse: 5′-actcgtcatactcctgcttgct-3′
